# Genetic Variations in the Promoter of the *APE1* Gene Are Associated with DMF-Induced Abnormal Liver Function: A Case-Control Study in a Chinese Population

**DOI:** 10.3390/ijerph13080752

**Published:** 2016-07-25

**Authors:** Zhimin Tong, Huanxi Shen, Dandan Yang, Feng Zhang, Ying Bai, Qian Li, Jian Shi, Hengdong Zhang, Baoli Zhu

**Affiliations:** 1 Kunshan Municipal Center for Disease Prevention and Control, Kunshan 215301, China; 79972002tzm@163.com (Z.T.); shenhuanxi@163.com (H.S.); sjian@163.com (J.S.); 2 Department of Environmental Genomics, Jiangsu Key Laboratory of Cancer Biomarkers, Prevention and Treatment, Cancer Center, Nanjing Medical University, Nanjing 211166, China; 3 Department of Integrated Management & Emergency Preparedness and Response, Jiangsu Provincial Center for Disease Prevention and Control, Nanjing 210009, China; Yangdan1997@163.com; 4 Institute of Occupational Disease Prevention, Jiangsu Provincial Center for Disease Prevention and Control, No.172 Jiangsu Road, Nanjing 210009, China; zhangfeng0401@163.com (F.Z.); baiy@jscdc.cn (Y.B.); hd-zhang@263.net (H.Z.); 5 The First People’s Hospital of Kunshan, Kunshan 215300, China; qianyingbingfeng@163.com

**Keywords:** *N*,*N*-dimethylformamide, *hOGG1*, *XRCC1*, *APE1*, polymorphism

## Abstract

Acute or long-term exposure to *N*,*N*-dimethylformamide (DMF) can induce abnormal liver function. It is well known that DMF is mainly metabolized in the liver and thereby produces reactive oxygen species (ROS). The base excision repair (BER) pathway is regarded as a very important pathway involved in repairing ROS-induced DNA damage. Several studies have explored the associations between *GSTM1*, *GSTT1*, *CYP2E1* polymorphisms and DMF-induced abnormal liver function; however, little is known about how common *hOGG1*, *XRCC1* and *APE1* polymorphisms and DMF induce abnormal liver function. The purpose of this study was to investigate whether the polymorphisms in the *hOGG1* (rs159153 and rs2072668), *XRCC1* (rs25487, rs25489, and rs1799782), *APE1* (rs1130409 and 1760944) genes in the human BER pathway were associated with the susceptibility to DMF-induced abnormal liver function in a Chinese population. These polymorphisms were genotyped in 123 workers with DMF-induced abnormal liver function and 123 workers with normal liver function. We found that workers with the *APE1* rs1760944 TG/GG genotypes had a reduced risk of abnormal liver function, which was more pronounced in the subgroups that were exposed to DMF for <10 years, exposed to ≥10 mg/m^3^ DMF, never smoked and never drank. In summary, our study supported the hypothesis that the *APE1* rs1760944 T > G polymorphism may be associated with DMF-induced abnormal liver function in the Chinese Han population.

## 1. Introduction

*N*,*N*-Dimethylformamide (DMF) is a colourless liquid organic solvent that is miscible with water and most organic solvents. DMF is widely used in industry, particularly in the manufacture of synthetic leather and resins. It was reported that China produces and consumes the largest amount of DMF in the world [1], therefore, many workers are exposed to DMF. Acute or long-term exposure to DMF can induce abnormal liver function, liver damage with steatosis, spotty necrosis, diffuse regeneration and even death [2,3,4,5,6]. DMF can easily be absorbed through oral, dermal, or inhalation exposure [7,8], after which it is mainly metabolized in the liver [8]. It is well known that cytochrome P450 family 2 subfamily E member 1 (CYP2E1) plays a key role in metabolizing DMF and thereby producing reactive oxygen species (ROS) [9]. Furthermore, the sustained ROS challenge aggravates liver cell viability. ROS are very harmful to liver cells because they can injure cellular DNA, proteins, and lipids [10,11]. Among all types of oxidative DNA damage, 8-hydroxydeoxyguanosine (8-OHdG) has been the main focus as a ubiquitous marker of oxidative stress because of its demonstrated mutagenic potential.

The base excision repair (BER) pathway is regarded as a very important pathway involved in repairing ROS-induced DNA damage [12,13]. Many proteins are involved in BER, however, the roles of human 8-oxoguanine DNA glycosylase (hOGG1), X-ray repair cross-complementing group 1 (XRCC1) and apurinic/apyrimidinic endonuclease-1 (APE1) have been well studied. hOGG1 is a protein that specifically repairs oxidative damage, primarily the 8-hydroxy-2’-deoxyguanosine DNA adducts resulting from ROS [14]; XRCC1 is a scaffolding protein that can interact with multiple enzymatic components at every stage of the repair pathway. Moreover, XRCC1 can repair single strand breaks resulting from the BER process [15]. APE1 is an essential protein that could excise the apurinic/apyrimidinic (AP) sites generated when glycosylases initiate the repair of a damaged base; APE1 also helps recruit DNA polymerase β (POL β) and facilitates further steps in the repair process [16]. 

Common single-nucleotide polymorphisms (SNPs) in the 8-oxoguanine glycosylase-1 (*OGG1*), the X-ray repair cross-complementing-1 (*XRCC1*), and the apurinic/apyrimidinic endonuclease-1 (*APE1*) genes in the BER pathway have been well studied for their influences on an individual’s sensitivity to the induction of DNA damage [17,18,19,20]; genetic variations in *hOGG1*, *XRCC1* and *APE1* could increase or decrease the risk of various cancers and noise-induced hearing loss [21,22,23,24]. Several studies have explored the associations between *GSTM1*, *GSTT1*, and *CYP2E1* polymorphisms and DMF-induced abnormal liver function [25,26,27,28]; however, little was known about the relation between common *hOGG1*, *XRCC1* and *APE1* polymorphisms and DMF-induced abnormal liver function. 

We genotyped 123 workers with DMF-induced abnormal liver function and 123 workers with normal liver function and compared the genotype frequencies between these two groups to investigate whether polymorphisms in the *hOGG1* (rs159153 and rs2072668), *XRCC1* (rs25487, rs25489, and rs1799782), and *APE1* (rs1130409 and 1760944) genes in human BER pathway were associated with susceptibility to DMF-induced abnormal liver function in a Chinese population.

## 2. Materials and Methods

### 2.1. Subjects

This study enrolled 123 workers with DMF-induced abnormal liver function and 123 workers with normal liver function from a synthetic leather factory in Kunshan (Jiangsu Province, China). All the subjects (age range, 23–60 years) were Han Chinese. DMF-exposed workers must have an occupational health examination once a year (the pre-occupational health examination was performed before the workers were employed and workers with an occupational contraindication were not employed), according to the Technical Specifications for Occupational Health Surveillance (2014) of China. The selected subjects had no medical factors associated with abnormal liver function, such as chronic hepatitis or fatty liver, and were not presently undergoing drug treatments. Moreover, the included subjects were not habitually exposed to other chemical factors associated with abnormal liver function (e.g., organic phosphorus, carbon tetrachloride, etc.). As the known non-occupational factors were excluded, it was presumed that the abnormal liver function observed in the subjects was mainly induced by DMF. Subjects who had smoked 100 cigarettes or more in their lifetime were defined as chronic smokers and the remaining subjects were defined as non-smokers. Subjects who consumed three or more alcohol drinks per week for at least one year were defined as chronic drinkers, and the rest were defined as non-drinkers. The demographic and occupational data (such as work history and DMF exposure time) were collected using structured questionnaires, and these questionnaires were administered in face-to-face interviews conducted by our topic-based group for each subject. The study was approved by the Institutional Review Board of Jiangsu Provincial Center for Disease Prevention and Control (JSJK2014-B030-02). Written informed consent was obtained from all participants.

### 2.2. Liver Function Assessment and Measurements of the Environmental DMF Concentrations

GE LOGIQ 400 (GE Company, Fairfield, CT, USA) was applied to distinguish parenchymal liver diseases, liver cirrhosis and fatty liver. Five millilitres of venous blood were donated by all the volunteers, and immediately centrifuged at 1600 *g* for 10 min (blood cells and serum were separated and stored at −80 °C). The biochemical measurements were collected using a Beckman AU 5800 (Beckman Coulter, Inc., Brea, CA, USA); alanine aminotransferase (ALT) levels of more than 40 U/L were defined as DMF-induced abnormal liver function (subjects were excluded who drank 3 days before the liver function assessment), otherwise ALT levels of no more than 40 U/L were defined as normal liver function. We named the “workers with DMF-induced abnormal liver function” and normal liver function as “cases” and “controls”, respectively. The DMF concentrations were measured strictly according to the Specifications of Air Sampling for Hazardous Substances Monitoring in the Workplace (GBZ 159-2004), and the 8 h-TWA (time-weighted average) concentration was calculated. The current threshold limit value (TLV) of DMF in the workplace is 20 mg/m^3^.

### 2.3. Genotyping of hOGG1, XRCC1 and APE1 Polymorphisms

Genomic DNA was isolated from the peripheral blood samples using a DNA extraction kit (TianGen, Beijing, China) according to standard procedures. The *hOGG1*, *XRCC1* and *APE1* polymorphisms were detected using the TaqMan SNP Genotyping assay and the 96-well ABI 7900HT Real Time PCR System (Applied Bio-systems, Foster City, CA, USA). The primer sequences and the probes were designed and manufactured by Nanjing Ji Ao Biological Technology Co., Ltd. (Nanjing, China). The final volume for each reaction was 10 μL, consisting of 0.25 μL of FAM-probe, 0.25 μL of HEX-probe, 0.5 μL of F-prime, 0.5 μL of R-prime, 2.5 μL of ddH_2_O, 5 μL of TaqMan Master Mix, and 10 ng of DNA. The PCR profile consisted of an initial denaturation step at 50 °C for 2 min and 95 °C for 10 min, and 40 cycles of 95 °C for 15 s and 60 °C for 1 min. All the fluorescence levels were detected using an 7900HT Real-Time PCR System (ABI, Waltham, MA, USA). The allele frequencies were determined using ABI SDS 2.3 software (Waltham, MA, USA). All the measurements were conducted according to the standard operating procedure, which was supplied by the biological technology company. 

Two polymorphisms (*hOGG1* rs2072668 and *APE1* rs1130409) could not be detected in most subjects, although three attempts were made. Therefore, only five polymorphisms of *hOGG1*, *XRCC1* and *APE1* were analysed in our study. For quality control, two people independently performed the genotyping in a blinded fashion. More than 10% of the genotypes were randomly chosen for confirmation, and the reproducibility was 100%.

### 2.4. Statistical Analysis

The data were analysed using SAS statistical software (version 9.1.3; SAS Institute, Cary, NC, USA). Continuous data were computed using the independent-sample two-sided *t* test. Categorical data were analysed with the two-sided χ^2^ tests. Medians and quartiles were used to describe the distribution of ALT (*p* < 0.05). Two-sided Wilcoxon rank sum test was used for comparing nonparametric values. Hardy-Weinberg equilibrium was tested using the goodness-of-fit χ^2^ test. Multivariate logistic regressions were used to calculate odds ratios (ORs) and 95% confidence intervals (95% CIs) to explore the associations between different genotypes with DMF-induced abnormal liver function susceptibility. Adjusted ORs and 95% CIs were computed by multivariate logistic regression adjusted for age, sex, smoking status, and drinking status. Furthermore, the stratification analyses were presented according to the subgroups of DMF exposure concentration, DMF exposure time, smoking status, and drinking status to estimate the different combinations of the *hOGG1*, *XRCC1* and *APE1* genetic variants between the cases and controls. All of the tests were two-sided, and a *p-*value < 0.05 was considered statistically significant.

## 3. Results 

### 3.1. Subjects’ Characteristics

The demographic and occupational characteristics of the 123 cases and 123 controls are presented in Table 1. No significant differences were found in the distribution of age, smoking status, drinking status, exposure concentration, or exposure time between the cases and controls. However, there were more females in the cases than controls (52.0% vs. 6.5%), the mean ALT value of the cases was approximately five-fold higher than the controls (53.4 ± 7.7 (U/L) vs. 11.7 ± 2.6 (U/L), *p* < 0.001). Although the alcohol consumption (less than 10 g per day) and the average pack-years (less than five cigarettes per day) of the subjects were less, we did not quantitatively analyse the effects of drinking and smoking.

### 3.2. Associations of the hOGG1, XRCC1 and APE1 Polymorphisms with the Susceptibility to Abnormal Liver Function

The observed frequencies of the *hOGG1*, *XRCC1* and *APE1* genotypes among the cases and controls and their associations with the risk of abnormal liver function are presented in Table 2. Four genotype frequencies (*XRCC1* rs25487, rs25489, rs1799782, and *APE1* rs1760944) conformed to Hardy-Weinberg equilibrium (*p* = 0.738, 0.721, 0.273, and 0.220, respectively). However, the *hOGG1* rs159153 genotype frequencies did not meet Hardy-Weinberg equilibrium (*p* = 0.001). The distributions of the *hOGG1* rs159153, *XRCC1* rs25487, *XRCC1* rs1799782, *XRCC1* rs25489 polymorphisms were not significantly different between workers with DMF-induced abnormal liver function and workers with normal liver function. However, for the *APE1* rs1760944 polymorphism, the TT, TG, and GG genotypes represented 28.5%, 43.9%, and 27.6% of the controls, respectively, and the TT, TG, and GG genotypes represented 42.3%, 41.5%, and 16.3% of the cases, respectively (*p* = 0.029). Multivariate logistic regression analyses were also used to explore the associations between these polymorphisms and the susceptibility to abnormal liver function. When we used the *APE1* rs1760944 TT genotype as the reference, we found that the *APE1* rs1760944 GG genotype could decrease the risk of abnormal liver function (Adjusted OR = 0.34, 95% CI = 0.14–0.82). When we combined the *APE1* rs1760944 TG and GG genotypes in a recessive model, we found that individuals with the *APE1* rs1760944 TG/GG genotypes had a reduced risk at abnormal liver function compared with those with the *APE1* rs1760944 TT genotype (Adjusted OR = 0.46, 95% CI = 0.25–0.87).

### 3.3. Stratification Analyses between the APE1 rs1760944 Polymorphism and the Risk of Abnormal Liver Function

The results of stratification analyses (TG/GG vs. TT) are shown in Table 3. We observed that the decreased risk was more evident in groups with <10 years of exposure (Adjusted OR = 0.27, 95% CI = 0.10–0.74), ≥10 mg/m^3^ DMF environmental exposure (Adjusted OR = 0.35, 95% CI = 0.14–0.85), the never smoking status (Adjusted OR = 0.42, 95% CI = 0.18–0.95), and the never drinking status (Adjusted OR = 0.38, 95% CI = 0.19–0.76), and the adjusted ORs in these groups were all less than 0.46.

## 4. Discussion

In this study, we explored the associations between *hOGG1*, *XRCC1* and *APE1* polymorphisms with the risk of DMF-induced abnormal liver function in a case-control study. We found that the *APE1* rs1760944 TG/GG genotypes conferred a significantly reduced risk of DMF-induced abnormal liver function compared with the *APE1* rs1760944 TT genotype. The reduced risk was more evident in the groups with <10 years of exposure, ≥10 mg/m^3^ DMF environmental exposure (Adjusted OR = 0.35, 95% CI = 0.14–0.85), the never smoking status (Adjusted OR = 0.42, 95% CI = 0.18–0.95), and the never drink status (Adjusted OR = 0.38, 95% CI = 0.19–0.76).

APE1 is a multifunctional enzyme involved in the BER pathway, which repairs oxidative base damage caused by endogenous and exogenous agents [29]. APE1 can incise the DNA 5’ at the AP sites; then, repair proceeds to the short-patch (only 1 nucleotide gap) or long-patch (≥2 nucleotides gap) subpathways of BER [30]. Polymorphisms in DNA repair genes have been shown to influence the activity DNA repair enzymes and may influence the susceptibility to various cancers [31,32]. The human *APE1* gene is located on chromosome 14q11.2-q12, which consists of five exons spanning 2.21 kb. In recent years, many studies have reported that the −656 T > G (rs1760944) polymorphism in the promoter of the *APE1* gene was associated with the risk of cancers [33,34]. 

Functional studies have shown that the *APE1* −656 G allele was associated with a reduced risk of lung cancer and cervical cancer compared with the *APE1* −656 T allele [34,35]. It was also reported that the *APE1* −656 T > G polymorphism (rs1760944) could affect the promoter activity of APE1 and had a protective effect on cancer risk [36]. Similarly, in our study, we found that the *APE1* rs1760944 polymorphism (−656 T > G) might reduce the risk of DMF-induced abnormal liver function in a Chinese population.

Generally speaking, if an individual is exposed to DMF for longer periods, the cumulative DMF exposure may be greater, and the subjects would be more susceptible to abnormal liver function. In our study, we found that workers with the *APE1* rs1760944 TG/GG genotypes in the <10 years of exposure group had a significantly decreased risk of abnormal liver function than subjects carrying the *APE1* rs1760944 TT genotype (Adjusted OR = 0.27, which is less than 0.46). In animal research, increased levels of ALT and AST were detected when the animal was exposed to DMF at concentrations of 200 ppm or more [37]. A human study also had been reported that DMF-induced abnormal liver function was positively associated with the DMF exposure concentration [4]. However, in our study, we observed that the protective effects of the *APE1* rs1760944 TG/GG genotypes were more significant only in the <10 years of exposure group (Adjusted OR = 0.35, less than 0.46). The DMF exposure concentration in this study was below the permissible concentration-time weighed average (PC-TWA); moreover, liver damage induced by low level DMF exposure is still controversial [1].

Smoking is being recognized as a potential environment contaminant and is linked to increased oxidative stress and inflammation in the liver tissue [38]. However, little is known about the relation between smoking and abnormal liver function in humans. In our study, we observed that the protective effect of *APE1* rs1760944 TG/GG was more evident among the never smoking subjects; more studies needed to be conducted to further validate this finding. Alcohol consumption was found to impact liver function in many studies [2,39]. Similarly, in our study, when the *APE1* −656 TG/GG genotype was combined with never drinking, the protective effect of *APE1* rs1760944 TG/GG was more significant (Adjusted OR = 0.38, less than 0.46). 

Compared to other studies, we were the first to investigate whether polymorphisms in genes in the BER pathway [*hOGG1* (rs159153 and rs2072668), *XRCC1* (rs25487, rs25489, and rs1799782), and *APE1* (rs1130409 and 1760944)] were associated with DMF-induced abnormal liver function. The cases and controls enrolled in this study were matched according to their demographic factors and were exposed to a similar occupational workplace. However, several limitations still existed in our study. First, the number of the subjects enrolled was small. Second, workers with abnormal liver function must be transferred to a no DMF exposure environment, according to the occupational prevention laws. Therefore, some susceptible subjects may not have been enrolled in our study. Third, the distribution of *GSTM1*, *GSTT1*, and *CYP2E1* polymorphisms in such population was not assessed and presented in our study.

## 5. Conclusions

In summary, a functionally significant rs1760944 T > G polymorphism in the *APE1* promoter region was identified in our study, which may contribute to the susceptibility to DMF-induced abnormal liver function in a Chinese population. We found that workers with the *APE1* rs1760944 TG/GG genotypes had a significantly reduced risk of abnormal liver function. This significantly reduced risk was more pronounced in <10 years of DMF exposure, ≥10 mg/m^3^ DMF exposure concentrations, never smoking, and never drinking subgroups. However, more subjects needed to be enrolled to validate these findings.

## Figures and Tables

**Table 1:** Demographic and occupational characteristics of the cases and controls.

Variables	Controls (*n* = 123)		Cases (*n* = 123)			*p* ^a^
	*n*	%	*n*	%		
Age (years)	42.1 ± 7.7		41.3 ± 8.0			0.410 ^b^
<35	23	18.7	23	18.7		0.606
35–45	54	43.9	61	49.6		
>45	46	37.4	39	31.7		
Sex						
Male	115	93.5	59	48.0		**<0.001**
Female	8	6.5	64	52.0		
Smoking status						
Never	82	66.7	92	74.8		0.161
Ever	41	33.3	31	25.2		
Drinking status						
Never	104	84.6	106	86.2		0.718
Ever	19	15.4	17	13.8		
Exposure concentration (mg/m^3^)	10.4 ± 5.7		9.3 ± 5.3			0.118 ^b^
<10	56	45.5	69	56.1		0.097
≥10	67	54.5	54	43.9		
Exposure time (years)	11.8 ± 8.3		12.7 ± 7.8			0.384 ^b^
<10	56	45.5	43		35.0	0.091
≥10	67	54.5	80		65.0	
ALT (U/L)	13 (11, 13)		49 (44, 55)			**<0.001** ^c^

^a^ Two-sided χ^2^ tests for the comparisons of the ages, exposure concentrations, exposure times and ALT levels between the cases and controls; ^b^ Two-sided *t* tests of the frequency distributions of selected variables between the cases and controls; ^c^ Two-sided Wilcoxon rank sum test for comparing the ALT values between the cases and controls.

**Table 2:** Associations of *hOGG1, APE1, XRCC1* polymorphisms with the risk of DMF-induced abnormal liver function.

Genotypes	Controls (*n* = 123)	Cases (*n* = 123)	Adjusted ^a^ OR (95% CI)	*p* ^b^
	*n* (%)	*n* (%)		
*hOGG1* rs159153				0.772
CC	5 (4.1)	7 (5.7)	1.00	
CT	19 (15.5)	21 (17.1)	0.64 (0.14–3.03)	0.722
TT	99 (80.4)	95 (77.2)	0.48 (0.13–1.88)	0.529
CT + TT	118 (95.9)	116 (94.3)	0.51 (0.13–1.94)	0.554
*XRCC1* rs 25487				0.077
GG	62 (50.4)	71 (57.7)	1.00	
GA	42 (34.2)	44 (35.8)	1.11 (0.58–2.13)	0.747
AA	19 (15.4)	8 (6.5)	0.48 (0.18–1.29)	0.024
GA + AA	61 (49.6)	52 (42.3)	0.91 (0.50–1.65)	0.250
Rs 1799782				0.585
CC	60 (48.8)	54 (43.9)		
CT	48 (39.0)	56 (45.5)	1.18 (0.62–2.28)	0.339
TT	15 (12.2)	13 (10.6)	1.03 (1.39–2.71)	0.929
CT + TT	63 (51.2)	69 (56.1)	1.14 (0.23–2.08)	0.443
Rs 25489				0.250
CC	96 (78.1)	98 (79.7)		
CT	22 (17.8)	24 (19.5)	1.37 (0.64–2.92)	0.840
TT	5 (4.1)	1 (0.8)	0.53 (0.06–4.76)	0.102
CT + TT	27 (21.9)	25 (20.3)	1.22 (0.59–2.50)	0.755
*APE1* rs1760944				**0.029**
TT	35 (28.5)	52 (42.3)		
TG	54 (43.9)	51 (41.5)	0.57 (0.29–1.14)	0.121
GG	34 (27.6)	20 (16.3)	**0.34 (0.14–0.82)**	**0.009**
TG + GG	88 (71.5)	71 (57.8)	**0.46 (0.25–0.87)**	**0.023**

^a^ Adjusted for age, sex, smoking status, and drinking status. ^b^ Two-sided χ^2^ test of the frequency distributions of selected variables between the cases and controls.

**Table 3:** Stratified analysis of the *APE1* rs1760944 genotypes (TG/GG vs. TT) associated with the risk of DMF-induced abnormal liver function.

Variables	Controls		Cases		OR (95% CI)	Adjusted OR (95% CI) ^a^	*p* ^b^
	TG/GG (88)	TT (35)	TG/GG (71)	TT (52)			
	*n* (%)	*n* (%)	*n* (%)	*n* (%)			
Exposure time (years)							
<10	39 (39.4)	17 (17.2)	19 (19.2)	24 (24.2)	**0.35 (0.15–0.79)**	**0.27 (0.10–0.74)**	**0.011**
≥10	49 (33.3)	18 (12.2)	52 (35.4)	28 (19.1)	0.68 (0.34–1.39)	0.72 (0.30–1.69)	0.290
Exposure concentration (mg/m^3^)							
<10	40 (32.0)	16 (12.8)	41 (32.8)	28 (22.4)	0.59 (0.28–1.24)	0.65 (0.25–1.66)	0.162
≥10	48 (39.7)	19 (15.7)	30 (24.8)	24 (19.8)	0.50 (0.23–1.05)	**0.35 (0.14–0.85)**	0.066
Smoking status							
Never	58 (33.3)	24 (13.8)	52 (22.9)	40 (23.0)	0.54 (0.29–1.01)	**0.42 (0.18–0.95)**	0.052
Ever	30 (41.7)	11 (15.3)	19 (26.4)	12 (16.6)	0.58 (0.21–1.58)	0.64 (0.22–1.86)	0.284
Drinking status							
Never	73 (34.8)	31 (14.8)	57 (27.1)	49 (23.3)	**0.49 (0.28–0.87)**	**0.38 (0.19–0.76)**	**0.014**
Ever	15 (41.7)	4 (11.1)	14 (38.9)	3 (8.3)	1.24 (0.24–6.58)	1.64 (0.28–9.57)	0.797

^a^ Adjusted for age, sex, smoking status, and drinking status; ^b^ Two-sided χ^2^ test of the frequency distributions of selected variables between the cases and controls.
